# Diagnostic accuracy in dermatology across skin tones: A systematic review

**DOI:** 10.1016/j.jdin.2026.07.007

**Published:** 2026-07-29

**Authors:** Jiayan Ding, Darryl Chin, Ellie Choi, Valencia Long

**Affiliations:** a Department of Medicine, Yong Loo Lin School of Medicine, National University of Singapore, Singapore; b Division of Dermatology, Department of Medicine, National University Hospital, Singapore

**Keywords:** dermatology, diagnostic accuracy, diagnostic error, diversity, epidemiology, inclusion, medical education, misdiagnosis, racial bias, review, sensitivity, skin of color, specificity

*To the Editor*: Skin tone can alter the visual presentation of dermatologic conditions and affect diagnostic recognition. This systematic review synthesizes evidence comparing diagnostic accuracy across skin tones.

Studies comparing diagnostic accuracy, or proportion of correct diagnoses, among physicians or medical students between skin of color (SoC) and light skin (LS) cases were included while those assessing only machine-learning performance or lacking an established reference diagnosis were excluded (Supplementary Methods, available via Mendeley at https://data.mendeley.com/datasets/ztnh4w2vzy/1).

PubMed and Embase were searched on June 1, 2026, using terms including “diagnostic accuracy” and “SoC” (Supplementary Tables II a and II b, available via Mendeley at https://data.mendeley.com/datasets/ztnh4w2vzy/1). This yielded 3724 articles, and 16 were eventually included (Fig 1). All studies were conducted in North America or Europe and majority assessed diagnostic accuracy via experimental formats using clinical images, vignettes, or multiple-choice questions; only 3 evaluated real-world clinical misdiagnosis rates (Table I). Given substantial clinical and methodological heterogeneity, findings were synthesized descriptively.

**Fig 1:**
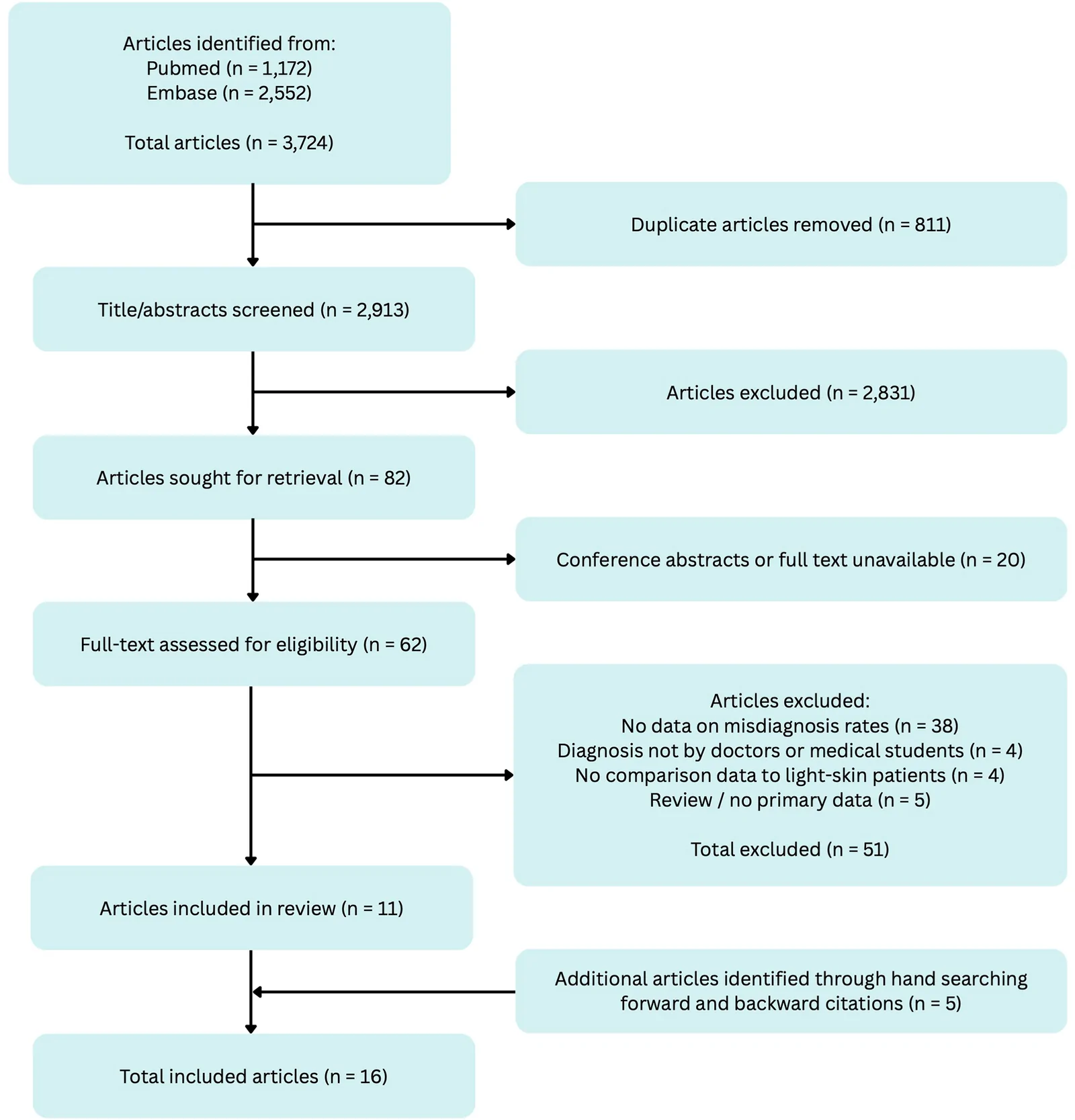
PRISMA flowchart of included articles.

**Table I:** Summary of included articles assessing diagnostic accuracy of LS vs SoC presentations

Characteristics	Number ofstudies	Remarks
Study participants		
Medical students	8	8 studies assessed the diagnostic performance of 1184 medical students in SoC cases as compared to LS, with studies assessing either short-term improvements directly post-intervention (*n* = 3) or as a one-time observation (*n* = 5)
Dermatologists	3	572 dermatologists were assessed across 3 studies, with one study assessing diagnostic performance in LS and SoC skin diseases pre- and post- exposure to support from AI
Dermatology residents	1	1 study assessed the diagnostic performance of 116 dermatology residents in LS and SoC skin diseases
GPs	4	1879 GPs and 10 senior GP trainees were assessed across 4 studies, with one assessing diagnostic performance in LS and SoC skin diseases pre- and post- exposure with support from AI, while another study assessed the diagnostic rates of melanoma in LS and SoC
Other physicians	2	2 studies assessed the variations in diagnostic performance between LS and SoC cases with dermatofibrosarcoma protuberans and other dermatological conditions in physicians from different specialties. This included 30 general surgeons, 30 surgical oncologists, and 158 physicians of unspecified specialty.
Study contexts		
Experimental settings	13	13 studies assessed diagnostic accuracy within experimental settings using image-based quizzes or multiple-choice questions, with or without case vignettes
Clinical misdiagnosis rate	3	3 studies assessed real-world clinical misdiagnosis rates
Study outcome: Differences in LS vs SoC diagnostic accuracy rates (GRADE certainty: very low)		
Overall worse diagnostic accuracy in SoC as compared to LS	11	1 study found that the top 1 diagnostic accuracy was significantly poorer in SoC even with support from AI for GPs, while the disparity in accuracy rates was not significant in dermatologists
		2 studies found poorer overall accuracy in SoC as compared to LS pre-intervention
		2 studies found higher real-world clinical misdiagnosis rates for SoC as compared to LS patients
		While the overall accuracy across 6 studies demonstrated a trend of poorer diagnostic accuracy in SoC, a significant reversal of this trend was observed in some studies for tinea versicolor, tinea capitis, jaundice, and urticaria, attributed to manifestations of pigmentary changes or increased prevalence in SoC
Overall better diagnostic accuracy in SoC as compared to LS	3	1 study found poorer confidence in SoC as compared to LS despite better diagnostic performance in SoC
		1 study reported significantly better diagnostic performance in SoC in 2025, after a curriculum update with significantly increased proportion of SoC images from 2019 to 2025
		1 study found poorer diagnostic performance for lupus in SoC, regardless of sex of the patients assessed in the case vignette
No significant differences in misdiagnosis between LS and SoC	2	1 study found overall no significant racial disparities in diagnostic accuracy, but with second-year medical students showing significantly better diagnostic accuracy in LS than SoC images compared to first-years
		1 study only assessed misdiagnosis in alopecia areata, finding no racial disparities in timing and frequency of misdiagnosis of alopecia areata

*AI*, Artificial intelligence; *GP*, general practitioner; *LS*, light skin; *SoC*, skin of color.

Nine studies reported overall lower diagnostic accuracy in SoC than LS images. Specifically, studies found reduced accuracy for SoC images in skin cancers, atopic dermatitis, herpes zoster, cellulitis, meningococcemia and Lyme disease. Conversely, similar or higher accuracy for SoC cases was reported in lupus, tinea versicolor, and tinea capitis, suggesting variations in diagnostic performance by morphology and prevalence. For example, higher diagnostic accuracy for tinea versicolor in SoC may reflect greater pigmentary changes, while higher recognition of tinea capitis, acne keloidalis nuchae and lupus may reflect greater familiarity from higher prevalence.^1^, ^2^, ^3^ Assessors’ own skin-type did not significantly affect diagnostic performance.

Two reported higher misdiagnosis rates among SoC patients, while 1 study found no racial disparities in timing or frequency of misdiagnosis.

A single study showed deep learning improved generalists’ accuracy, but less for SoC than LS images, widening the diagnostic gap.^4^

Overall, diagnostic accuracy was lower for SoC than LS presentations in 11 studies, higher for SoC than LS presentations in 3 studies, and not significantly different in 2 studies. However, these findings should be interpreted cautiously given the overall very low GRADE certainty owing to high heterogeneity, variable risk of bias (Supplementary tables V a-f, available via Mendeley at https://data.mendeley.com/datasets/ztnh4w2vzy/1), and limited real-world studies.

Critically, no studies were conducted in SoC-majority settings, limiting the ability to disentangle inadequate exposure, intrinsic diagnostic difficulty, and fundamentally unreliable frameworks (eg inflammation = rubor).^2^ Educational interventions improving exposure to disease morphology in SoC patients appeared beneficial in experimental settings,^5^ but longitudinal follow-up is needed to determine whether early gains translate into sustained real-world benefits. Human-AI collaboration could be another useful adjunct moving forward, with representative databases being key to building equitable algorithms.^4^

Further research should assess real-world misdiagnosis rates across other continents, focusing on their material impact on disease outcomes. Longitudinal studies are also needed to evaluate how diagnostic accuracy changes over time with shifting demographics. Education strategies can also place greater emphasis on correcting inbuilt terminologies centered around LS presentations.

## Declaration of generative AI and AI-assisted technologies in the writing process

During the preparation of this work, the author(s) used OpenEvidence for searching for additional studies that may have been missed in the initial search. These studies were independently verified by at least 2 independent reviewers, and any discrepancies were resolved through discussion, as per the study’s protocols. The author(s) reviewed and edited the output as needed and take full responsibility for the content of the published article.

## Conflicts of interest

None disclosed.
